# Mucosal snare resection-endoscopic submucosal excavation for gastric submucosal tumors: a retrospective study (with video)

**DOI:** 10.3389/fonc.2025.1534196

**Published:** 2025-01-24

**Authors:** Wei Wei, Xiaolong Zheng, Yongli Ye, Hongxia Li, Yiping Hong, Jianting Cai

**Affiliations:** ^1^ Department of Gastroenterology, The Second Affiliated Hospital, College of Medicine, Zhejiang University, Hangzhou, China; ^2^ Department of Gastroenterology, The Affiliated Jinhua Hospital, Zhejiang University School of Medicine, Jinhua, China

**Keywords:** submucosal neoplasms, ESE, MSR-ESE, endoscopic surgery, efficacy and safety

## Abstract

**Aims:**

This study aimed to compare the clinical outcomes of patients with submucosal tumors treated with endoscopic submucosal excavation (ESE) and those treated with mucosal snare resection-endoscopic submucosal excavation (MSR-ESE).

**Methods:**

We retrospectively analyzed clinical data from patients who underwent ESE or MSR-ESE at the Second Affiliated Hospital of Zhejiang University School of Medicine between January 2023 and January 2024. Factors such as operation time, intraoperative perforation, postoperative adverse events, postoperative fasting time, antibiotic use, hospitalization duration, costs, and pathological diagnosis were compared between the two procedures.

**Results:**

A total of 180 patients with submucosal tumors were included in this study. The MSR-ESE group consisted of 75 patients (41.7%), while the ESE group had 105 patients (58.3%). Propensity score matching (PSM) showed no significant differences in postoperative antibiotic use, fasting time, or intraoperative perforation between the two groups (*P*>0.05). However, the MSR-ESE group demonstrated shorter operation and hospitalization times, lower hospitalization costs, and a reduced incidence of postoperative peritonitis (*P*<0.05). Multivariate logistic regression analysis identified operation time as an independent risk factor for unplanned intraoperative perforation, with the likelihood of perforation increasing significantly as operation time increased (*P*=0.007, OR=1.015, 95% CI, 1.004 to 1.026).

**Conclusion:**

MSR-ESE outperforms ESE with shorter operation times, lower costs, and fewer postoperative complications, making it a safe and effective treatment for gastric submucosal tumors.

## Introduction

Submucosal tumors (SMTs) of the gastrointestinal tract are elevated lesions originating from the muscularis mucosa, submucosa, or muscularis propria, including mesenchymomas, leiomyomas, lipomas, and neurogenic tumors (1). The esophagus and stomach are the primary locations for SMTs in the upper gastrointestinal tract (2). With the advent of endoscopy and endoscopic ultrasonography (EUS), the detection rate of SMTs has significantly increased. Approximately 0.76% of patients undergoing endoscopy are diagnosed with SMTs (3). Endoscopic treatment offers advantages over laparotomy, such as reduced invasiveness and lower costs.

Endoscopic treatments for submucosal tumors in the upper gastrointestinal tract include methods like endoscope band ligation (EBL), endoscopic submucosal dissection (ESD), endoscopic submucosal excavation (ESE), endoscopic full-thickness resection (EFTR), and submucosal tunneling endoscopic resection (STER) (4–6). ESE evolved from ESD to deal with deeper tumors. ESE surgery employs painless gastroscopic instruments to incise the gastric wall mucosa in the affected area, thereby fully exposing the lesion. The tumor is then entirely excised, and specialized titanium clips are utilized to seal the resulting wound. ESE surgery stands out as it can completely remove larger lesions while preserving the integrity of the gastric wall, achieving a 100% complete resection rate for tumors ≤1.5 cm (7).

Traditionally, these methods involve submucosal injection to separate the mucosa from the muscularis propria. However, submucosal injections can increase the time spent searching for tumors, prolonging the operation and raising the risk of adverse events. To address this issue, mucosal snare resection-endoscopic submucosal excavation (MSR-ESE) was developed. MSR-ESE is an improved method of ESE. All SMTs suitable for ESE surgery can be operated with MSR-ESE.

This study aims to compare the clinical efficacy of ESE and MSR-ESE in treating submucosal tumors.

## Methods

### Patients

This retrospective study included patients with gastric submucosal tumors who received traditional ESE and MSR-ESE at the Second Affiliated Hospital of Zhejiang University School of Medicine from January 2023 to January 2024. Diagnosis was confirmed through endoscopic ultrasonography and abdominal CT examination. All SMTs were diagnosed as gastrointestinal stromal tumors (GISTs) or other tumors with a tendency for malignant transformation through tissue biopsy. This included benign tumors smaller than 1 cm in size, which the patients strongly requested to have resected. Surgeries were conducted by experienced physicians at our institution. There are 216 patients in total. All patients followed the principles of the Declaration of Helsinki, and the study protocol was approved by the Ethics Committee of the Second Affiliated Hospital of Zhejiang University School of Medicine (2024-0226).

### Inclusion and exclusion criteria

**Figure 1:**
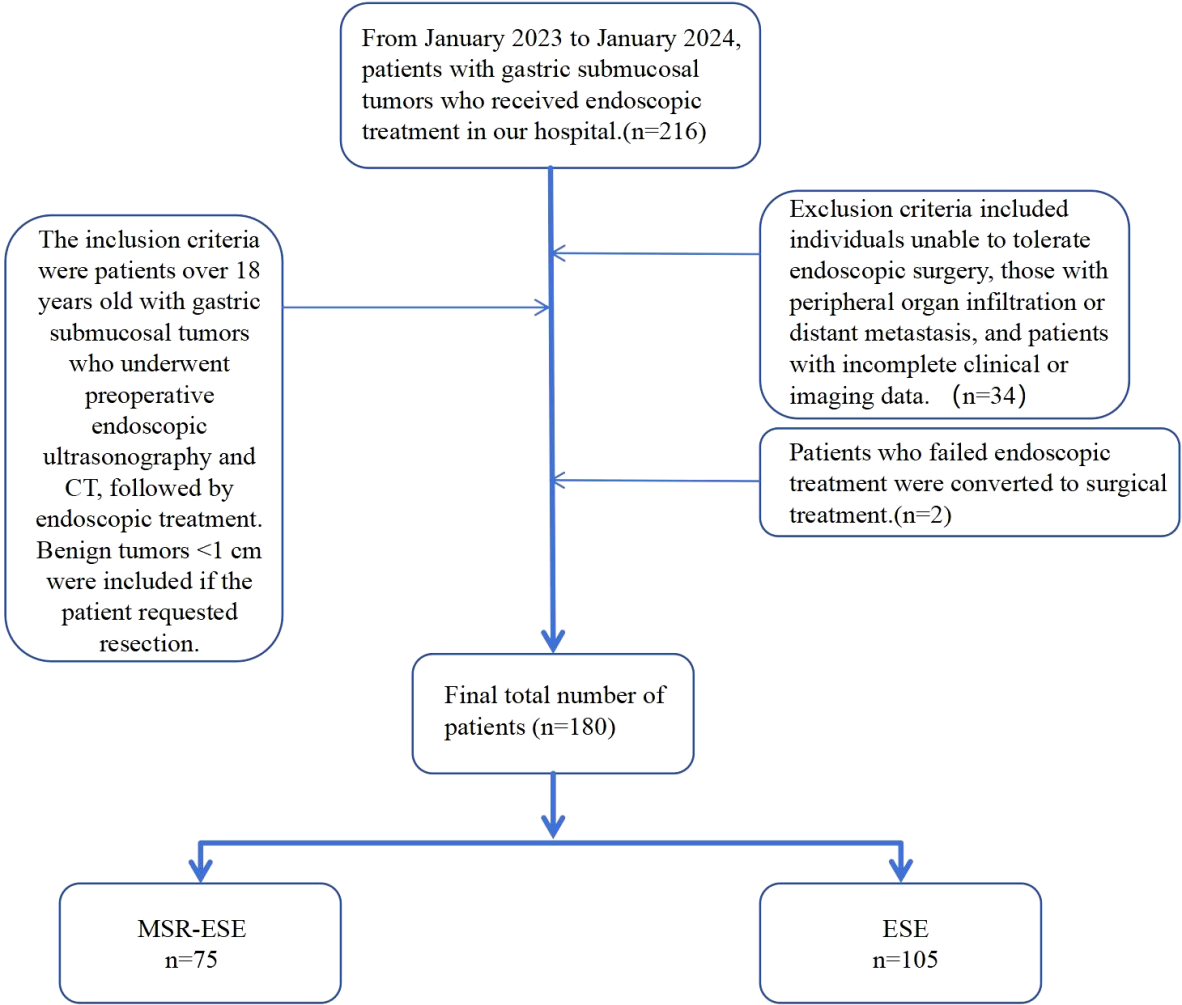
The research flow chart.

The inclusion criteria were patients aged over 18 years with gastric submucosal tumors who underwent endoscopic ultrasonography and abdominal contrast-enhanced CT prior to surgery and received endoscopic treatment at our hospital. Benign tumors <1 cm in diameter were included if the patient strongly requested resection. Exclusion criteria included individuals unable to tolerate endoscopic surgery, those with peripheral organ infiltration or distant metastasis, and patients with incomplete clinical or imaging data. After screening, 34 patients were excluded, and 2 with poor endoscopic outcomes were converted to surgery. Ultimately, 180 patients were included in the analysis (**Figure 1**).

### Surgery technique of MSR-ESE and ESE

Endoscopic submucosal excavation (ESE) involves the insertion of an endoscope into the upper gastrointestinal tract through the mouth to identify the lesion. A marker is burned at the lesion’s edge, followed by an injection of glycerol-fructose under the mucosa. The surface mucosa is then cut using the Dual knife, exposing the tumor with the help of both the Dual knife(Olympus KD-650L) and IT knife(Olympus KD-611L). The lesions are methodically peeled off and completely excavated along the tumor’s edge at its source level. Hemostatic treatment is applied to the wound, which is then closed using titanium clips (8).

**Figure 2:**
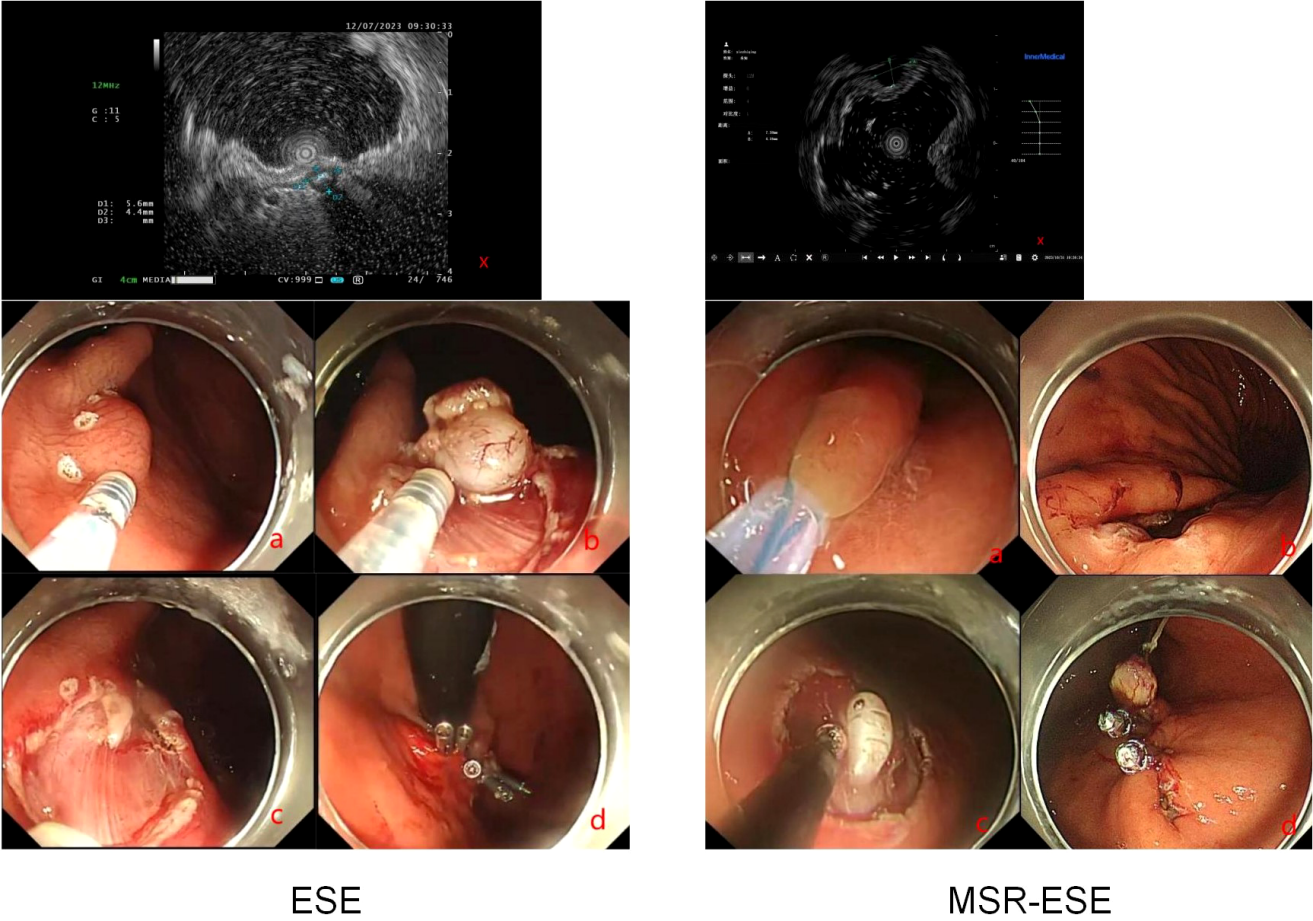
MSR-ESE and ESE surgical procedures. X: Endoscopic ultrasound. **(A–D)**: Demonstration of surgical procedures.

Mucosal snare resection-endoscopic submucosal excavation (MSR-ESE) begins with enclosing the surface mucosa of the lesion with a snare and performing electrocoagulation excision without prior submucosal injection. As the lesion is gradually exposed, it is completely dissected along its edge and above the muscularis propria by an insulation-tipped knife(Olympus KD-611L). After complete resection and retrieval of the lesion, the mucosal incision is closed tightly with metal clips (9)(**Figure 2**).

Postoperative treatment involves strict dietary restrictions, administration of proton pump inhibitors to protect the gastric mucosa, and fluid resuscitation. Monitoring the patient’s cardiac, hepatic, and renal functions, as well as vital signs, is crucial to detect any signs of bleeding, fever, perforation, or peritonitis. Diet should be gradually reintroduced based on the patient’s clinical status.

### Clinical data collection

Baseline data, such as surgical method, gender, age, BMI, and various blood parameters, were collected from the hospital’s medical record system. Short-term outcomes, including operative time, intraoperative bleeding, postoperative adverse events, antibiotic use, ICU admission, Postoperative fasting time and hospitalization costs, were also recorded. The operation time was defined as the time spent in the entire process from the insertion of the endoscope into the patient’s body to the completion of the operation, closure of the gastric wall wound, and extraction of the endoscope, and was recorded in minutes. Hospitalization expenses include the sum of all medical-related expenses for patients from admission to discharge, covering surgical expenses, drug expenses, examination expenses, and nursing expenses during hospitalization. The expense data comes from the hospital’s electronic medical record system, and the unit of amount is Yuan. Postoperative adverse events include postoperative abdominal pain, postoperative fever, postoperative bleeding, and postoperative peritonitis. Clinical efficacy was evaluated based on operation time, intraoperative perforation, surgical cost, hospital stay, and postoperative adverse events.

### Clinicopathological type and risk classification

We examined the clinicopathological types of specimens from all patients and performed risk classification. We analyzed the clinicopathological types of specimens and performed risk classification for all patients. Gastric submucosal tumors were categorized as benign or malignant based on origin and histology, including stromal tumors, leiomyomas, schwannomas, ectopic pancreas, neuroendocrine tumors, glomus tumors, calcifying fibromas, and cysts (10). Using the modified NIH 2008 criteria, stromal tumors were further classified by tumor size, mitotic index, and primary location into very low, low, intermediate, and high-risk groups (11).

### Statistical analysis

Statistical analysis was conducted using SPSS software. Independent samples t-test and paired-sample t-test were used for component difference analysis. For categorical data, the chi-square test and McNemar test were employed. Propensity score matching (PSM) was conducted to mitigate confounding variables. Matched baseline information included gender, age, BMI, underlying diseases, white blood cell count, hemoglobin, platelet count, total bilirubin, direct bilirubin, alanine aminotransferase, aspartate aminotransferase, γ-glutamyl-transpeptidase, alkaline phosphate, serum creatinine, prothrombin time, carcinoembryonic antigen. Logistic regression was utilized to examine the risk factors associated with passive perforation. A significance level of *P*<0.05 was considered statistically significant.

## Results

### Patients

The study included 180 patients with gastrointestinal submucosal tumors (SMT) who underwent endoscopic treatment at our hospital. Of these, 75 patients (41.7%) underwent MSR-ESE, while 105 patients (58.3%) underwent ESE. Among the patients, 62 (34.4%) were aged 60 years or older, and 51 (28.3%) were male. Additional baseline characteristics are detailed in **Table 1**.

**Table 1:** Baseline table.

Characteristics	Overall (N=180)
group (%)	
ESE	105(58.3%)
MSR-ESE	75(41.7%)
outcome (disease cure, %)	180(100.0%)
sex(%)	
Female	129(71.7%)
Male	51(28.3%)
age(%)	
<60	118(65.6%)
≥60	62(34.4%)
BMI, kg/m2	23.23 ± 3.03
hypertension or diabetes(%)	69(38.3%)
WBC	5.95 ± 2.05
Hb(g/L)	131.76 ± 14.69
PLT	206.16 ± 47.45
TB(umol/L)	12.88 ± 4.76
DB (umol/L)	2.35 ± 0.92
ALT	21.29 ± 12.79
AST	23.09 ± 7.38
GGT	25.72 ± 24.23
ALP	74.21 ± 18.04
Cr(umol/L)	64.90 ± 13.94
PT	12.85 ± 0.56
CEA	2.05 ± 1.00
intraoperative bleeding(%)	3(1.7%)
postoperative abdominal pain(%)	29(16.1%)
Postoperative fever(%)	16(8.9%)
Postoperative antibiotics(%)	126(70.0%)
Postoperative fasting time(h)	39.79(± 22.09)
Postoperative ICU(%)	1(0.6%)
postoperative bleeding(%)	2(1.1%)
postoperative hospital stay(day)	3.48 (± 1.61)
Hospital costs(Yuan)	19808.78 ± 5127.18
intraoperative perforation (%)	65(36.1%)
operation time(min)	60.56 ± 31.40

### Baseline characteristics after PSM

After propensity score matching (PSM), there were no significant differences in the baseline data between the two groups (*P* > 0.05), as shown in **Table 2**.

**Table 2:** Baseline and outcomes before and after PSM.

Baseline	Characteristics	Before PSM	After PSM
		MSR-ESE(75) ESE(105) p value	MSR-ESE(61) ESE(61) p value
	Sex	0.45	1
	Female	56(74.7%) 73(69.5%)	45(74%) 46(75%)
	Male	19(25.3%) 32(30.5%)	16(26%) 15(25%)
	Age	0.71	0.86
	<60	48(64%) 70(66.6%)	40(66%) 38(62%)
	≥60	27(36%) 35(33.4%)	21(34%) 23(38%)
	BMI, kg/m2	23.2 ± 2.8 23.3 ± 3.2 0.766	23.1 ± 2.8 22.9 ± 3.1 0.657
	hypertension or diabetes	28(37.3%) 41(39%) 0.816	23(38%) 20(33%) 0.728
	WBC(10^9/L)	6.2 ± 2.2 5.8 ± 1.9 0.17	6 ± 1.9 5.9 ± 1.9 0.877
	Hb(g/L)	132 ± 15 131 ± 15 0.859	131 ± 14 131 ± 13 0.959
	PLT	205.7 ± 42.6 206.5 ± 50.9 0.916	205.8 ± 43.3 200 ± 53.8 0.512
	TB(umol/L)	12.9 ± 4.7 12.9 ± 4.9 0.974	12.6 ± 4.4 13 ± 4.4 0.58
	DB(umol/L)	2.4 ± 0.8 2.4 ± 1 0.984	2.3 ± 0.8 2.4 ± 0.9 0.487
	ALT	22 ± 10 21 ± 14 0.53	21 ± 9 19 ± 13 0.348
	AST	23.6 ± 5.5 22.7 ± 8.5 0.414	22.9 ± 5 22.2 ± 7.6 0.518
	GGT	23.1 ± 17.8 27.6 ± 27.9 0.185	20.8 ± 7.8 20 ± 10 0.668
	ALP	75.8 ± 17.5 73.1 ± 18.4 0.334	73.3 ± 15.2 73.6 ± 17.8 0.929
	Cr(umol/L)	65 ± 15.9 64.8 ± 12.4 0.934	64.9 ± 16.7 65.8 ± 10.5 0.704
	PT(s)	12.8 ± 0.5 12.9 ± 0.6 0.435	12.9 ± 0.5 12.9 ± 0.6 0.768
	CEA	2.3 ± 1 1.9 ± 1 0.024	2.1 ± 0.9 2.1 ± 1 0.695
Outcomes	Characteristics	Before PSM	After PSM
		MSR-ESE(75) ESE(105) p value	MSR-ESE(61) ESE(61) p value
	intraoperative bleeding	0 3(2.9%) 0.14	0 2(3.3%)
	postoperative abdominal pain	6(8%) 23(21.9%) 0.012	5(8.2%) 13(21.3%) 0.077
	Postoperative fever	5(1.3%) 11(10.5%) 0.376	4(6.6%) 5(8.1%) 1
	postoperative bleeding	0 2(1.9%) 0.229	0 1(1.6%)
	Postoperative antibiotics	49(65.3%) 77(73.3%) 0.248	39(64%) 40(66%) 1
	Postoperative fasting time(h)	40.2 ± 19.4 39.5 ± 23.9 0.817	38.2 ± 18.7 39 ± 25.8 0.739
	Postoperative ICU	1(1.3%) 0 0.417	0 0
	postoperative hospital stay(day)	3.2 ± 1.1 3.7 ± 1.9 0.056	3.1 ± 1.1 3.7 ± 2.2 0.04
	Hospital costs(Yuan)	17590 ± 3956 21393 ± 5294 0.000	17200 ± 3847 21743 ± 5417 0.000
	intraoperative perforation	22(29.3%) 43(41%) 0.11	14(23%) 22(36%) 0.2
	operation time(min)	32.4 ± 15.6 80.7 ± 23.3 0.000	31.4 ± 15 81.3 ± 26 0.000
	clinical outcome	75(100%) 105(100%)	61(100%) 61(100%)

### Outcomes

The study found that the MSR-ESE group had significantly shorter operation times, postoperative hospitalization times, lower rates of postoperative peritonitis, and lower hospitalization costs compared to the ESE group (*P*< 0.05). These results indicate that MSR-ESE is less invasive than ESE. Detailed outcomes are presented in **Table 2**.

### Univariate and multivariable logistic regression analysis of passive perforation

Univariate logistic regression analysis assessed the risk of unplanned perforation in the two groups. The results indicated that operation time (*P* = 0.007, OR = 1.015, 95% CI, 1.004 to 1.026) was a potential risk factor for passive perforation. Considering that Hb and BMI have an impact on surgical outcomes (12), we chose to include them in multivariate analysis. Subsequent multivariate logistic regression analysis using the Backward: LR method confirmed that operation time (*P* = 0.007, OR = 1.015, 95% CI, 1.004 to 1.026) remained an independent risk factor. These findings are summarized in **Table 3**.

**Table 3:** Logistic regression analysis of intraoperative perforation.

Risk factors	Univariate analysis	Multivariable analysis	
	OR (95% CI) p value	OR (95% CI) p value	
surgical approach	1.911 (0.969-3.769) 0.062		
Sex	0.725 (0.360-1.461) 0.369		
Age	0.768 (0.393-1.501) 0.440		
Hypertension or diabetes	0.714 (0.369-1.382) 0.317		
Hb	1.013 (0.991-1.036) 0.235	1.012 (0.99-1.035)	0.282
BMI	0.967 (0.869-1.075) 0.535	0.936 (0.833-1.052)	0.269
Operationtime (min)	1.015 (1.004-1.026) 0.007	1.015 (1.004-1.026)	0.007

### Clinicopathological types and risk classifications

Among all submucosal tumors, there were 101 cases (56.1%) of stromal tumors, 65 cases (36.1%) of leiomyomas, 7 cases (3.9%) of schwannoma, and 4 cases (2.2%) of ectopic pancreas. Stromal tumors were more prevalent in the fundus of the stomach (n = 46, 45.5%), while leiomyomas were more common in the upper anterior wall of the gastric body (n = 41, 63%).

Rare types included neuroendocrine tumors (n = 1), glomus tumors (n = 1), and calcifying fibromas (n = 1), each found in different parts of the stomach (**Table 4**). In terms of risk classification of stromal tumors, 94 cases (93.07%) were classified as very low risk, 5 cases (4.95%) as low risk, and 2 cases (1.98%) as high risk. Histological margin results of all specimens were negative.

**Table 4:** Anatomical distribution of different pathological tissue types.

anatomical parts,n=180	Tumor type						
	Stromal tumor	leiomyoma	Schwannoma	Ectopic pancreas	neuroendocrine tumors	glomus tumor	calcified fibroma
fundus of stomach(46)	41(89.1%)	5(10.9%)					
the anterior wall in the upper gastric body(41)	17(41.5%)	24(58.5%)					
the posterior wall in the upper gastric body (34)	15(44.1%)	18(53%)		1(2.9%)			
anterior wall of the gastric antrum(4)	4(100%)						
the posterior wall of the gastric antrum (3)	1(33.3%)			1(33.4%)		1(33.3%)	
The junction of gastric antrum and gastric body(3)	1(33.3%)		2(66.7%)				
middle part of gastric body(14)	7(50%)	5(35.8%)	1(7.1%)	1(7.1%)			
lower body of stomach(4)			3(75%)				1(25%)
Upper lesser curvature of gastric body(9)	3(33.3%)	4(44.5%)	1(11.1%)	1(11.1%)			
lower curvature of gastric body(4)	4(100%)						
cardia(9)	3(33.3%)	6(66.7%)					
Greater curvature of upper gastric body(8)	5(62.5%)	3(37.5%)					
The junction of the fundus and the body of the stomach.(1)					1(100%)		

## Discussion

The stomach is a common site for submucosal tumors (SMT). Typically, patients with SMTs smaller than 2 cm do not exhibit noticeable symptoms. However, as tumors grow, symptoms such as bleeding and obstruction may manifest (13). With advancements in endoscopic technology, the detection rate of SMTs is increasing, with approximately one case found in every 300 endoscopic examinations (14). Endoscopic treatment offers efficiency and cost advantages over traditional laparotomy, preserving much of the stomach’s structure and enhancing the patient’s quality of life (15, 16).

In clinical practice, a combination of regular endoscopy, endoscopic ultrasound (EUS), and CT scans can be used to grade and assess lesions, guiding the selection of appropriate treatment methods. While ESE can enhance resection rates and reduce adverse events, its prolonged operation time and higher costs present challenges. Our study demonstrates that MSR-ESE significantly decreases operation time, hospitalization duration, and costs, while also lowering the risk of postoperative peritonitis.

MSR-ESE further shortens operation time. In ESE surgery, submucosal injection of glycerol-fructose is performed to separate the mucosa and muscularis propria, and then the tumor is separated (8). However, after submucosal injection, the dissection area increases, and when the tumor is smaller than 1 cm, its location may change, requiring more time to differentiate (18). Not only that, submucosal injection can cause mucosal edema, making wound closure difficult.MSR-ESE does not involve submucosal injection but directly excises the surface mucosa with a snare (19), which can better locate the tumor through a direct incision, at the same time, the snare will not cause tumor displacement, thus shortening the operation time, MSR-ESE has obvious advantages when processing small size SMT (such as <1 cm). It is noteworthy that while MSR-ESE demonstrates a shorter operation time compared to ESE, logistic regression analysis indicates that it is not a protective factor against accidental perforation during surgery. This finding may be attributed to individual variability and the limited number of cases. Although MSR-ESE reduces operation time, the risk of perforation is influenced by complex factors such as tumor location, size, and the degree of adhesion to surrounding tissues. Tumors in challenging locations may require more meticulous techniques, and those deeply embedded in the muscularis propria with strong adhesions to adjacent tissues can further elevate the risk of perforation. Additionally, as MSR-ESE is a relatively new technique with a small case volume, potential differences may not yet reach statistical significance. In conclusion, MSR-ESE effectively shortens operation time and provides valuable insights into preoperative perforation risk, contributing to improved surgical safety.

Medical costs are a significant concern that cannot be overlooked. Our study demonstrates that MSR-ESE leads to reduced hospitalization costs. Compared with ESE, MSR-ESE does not require double knives, thus reducing the cost of surgical consumables. This approach effectively addresses clinical issues while also decreasing expenses (19). Additionally, patients undergoing MSR-ESE generally experience shorter hospital stays compared to those undergoing ESE. Similar findings were observed in another study on submucosal injection ESE (17). This may be attributed to the fact that MSR-ESE avoids submucosal injection, effectively reducing mucosal surface tension and edema around the lesion. This facilitates better wound closure and promotes the healing process (20), which is associated with local vascular remodeling and improved blood flow (21). Moreover, the shorter operative time speeds up wound healing. Traditionally, oral feeding is initiated after the restoration of intestinal function, indicated by signs like flatulence and defecation. However, studies have suggested that early oral feeding following gastrointestinal surgery can reduce hospitalization duration without increasing adverse events (22). Therefore, when feasible and appropriate, early initiation of oral feeding is preferred. Another factor influencing hospital costs and length of stay is the use of postoperative antibiotics. Previous clinical studies have indicated that, in addition to treatments such as percutaneous endoscopic gastrostomy and esophageal sclerosis, prophylactic antibiotics may not significantly affect the incidence of postoperative endocarditis (23). Conversely, another study demonstrated that prophylactic antibiotics reduced the incidence of postoperative adverse events in patients undergoing endoscopic surgery (24). Considering the age and underlying conditions of the patients, although hospitalization costs may increase, antibiotics—specifically second-generation cephalosporins like cefuroxime—were administered to 126 patients (70.0%) post-surgery to prevent infection and reduce hospitalization duration. While there are concerns regarding antibiotic resistance, appropriate antibiotic prophylaxis can substantially mitigate complications and shorten hospital stays, thereby economically justifying the additional treatment costs associated with infection prevention.

SMTs are often deeply embedded in the muscularis propria and adhere tightly to surrounding tissues (25), making perforation a common postoperative complication requiring timely detection for optimal outcomes. In this study, intraoperative perforation occurred in 65 cases (36.1%) and was promptly managed with clipping. Active perforation was performed in cases with tumors showing predominant extraluminal growth on abdominal CT (n=10, 5.6%) and was not classified as an adverse event. Operation time was identified as an independent risk factor for passive perforation, consistent with prior studies showing increased risk with procedures exceeding 2 hours (26). Selecting appropriate surgical methods may reduce perforation rates (**Table 5**). Although most perforations resolve with conservative treatment (27), delayed recognition or failed management can lead to peritonitis symptoms, such as abdominal pain and fever, progressing to bacteremia or complicated intra-abdominal infection (cIAI) (28), requiring endoscopic suturing or surgical intervention (29). For patients with a history of endoscopic treatment, symptoms such as abdominal pain or tenderness, peritoneal irritation, and fever may occur after surgery. Blood inflammation indicators increase, and postoperative abdominal X-rays and CT may reveal free gas in the abdominal cavity or obvious defects in the digestive tract, which can be diagnosed as postoperative peritonitis (30). Our study demonstrated that MSR-ESE significantly reduced the risk of postoperative peritonitis compared to ESE (**Table 6**). However, due to the small sample size and lack of multicenter data, these findings should be interpreted cautiously. Perforation can lead to prolonged fasting, extended hospitalization, and increased costs, making it essential to account for this risk during lengthy surgeries. Interestingly, no correlation with BMI was observed, differing from ESD studies where lower perforation rates were reported in obese patients (31). This inconsistency may reflect differences in surgical techniques and sample sizes. Additionally, intraoperative and postoperative bleeding rates were comparable between the two groups.

**Table 5:** Previous studies on endoscopic surgery for perforation.

Author	Year	Sample size	Surgical approach	Outcomes
Chunyan Zeng	2019	12	DFT-ESE	There was no further bleeding or perforation after endoscopic closure of the perforation or wound after dental floss traction DFT-ESE, and no recurrence was found at follow-up.
Chen Du	2017		STER	By creating tunnels, STER maintains the integrity of the mucosa. This promotes wound healing and reduces infection rates.
Ping-Hong Zhou	2011	26	EFR	The key to EFR surgery is to successfully close the wall defect after resection. Failure to close the gap may lead to the occurrence of gastrointestinal fistula and abdominal infection.
Quan-Lin Li	2015	32	STER	The mucosal incision of STER is not in the same place as the tumor resection, which maintains the integrity of the gastrointestinal mucosa. Even if the lesion originates deep in the muscle layer, perforation will not occur. Therefore, STER may reduce postoperative gastrointestinal leakage. and risk of secondary infection.
Jia Liu	2021	397	ESE	Inexperience of doctors is a risk factor for difficult ESE (operative time ≥90 minutes), and poor exposure of the submucosal field is a problem for new doctors.
Qiang Zhang	2016	9	EMSLD	EMSLD preserves the mucosa above the tumor and can effectively reduce suture tension. When a perforation occurs, it can also be closed by retaining the mucosa.

**Table 6:** Postoperative peritonitis symptoms of MSR-ESE and ESE.

eSurgical approach	MSR-ESE(N=22)	ESE(n=43)	P
postoperative peritonitis	4(18.2%)	24(55.8%)	0.004
Postoperative antibiotics	21(95.5%)	40(93%)	1

The distribution of SMT sites correlates with their pathological types. Among 180 samples, stromal tumors were the most common, followed by leiomyomas, consistent with previous studies (32). Stromal tumors, originating from gastrointestinal stromal cells (Cajal cells), can occur throughout the digestive tract, with a higher prevalence in the gastric fundus (33). Gastric leiomyomas were predominantly located on the anterior and posterior walls of the upper gastric body, while recent research identified the cardia as the most frequent site (34). Due to their proximity, the cardia and adjacent areas are considered high-risk regions for gastric leiomyomas. Pathological risk assessment is essential for guiding treatment and prognosis, particularly for gastrointestinal stromal tumors (GIST) with malignant potential (35). Most stromal tumors in this study were low risk, with only 2 patients exhibiting high malignant potential, requiring further evaluation via EUS and possible surgery or chemotherapy (36).

This study provides the first comparative analysis of MSR-ESE and ESE but has several limitations. Despite using PSM to balance baseline data, retrospective studies may introduce selection bias, highlighting the need for prospective validation. The small sample size limits comprehensive analysis of surgical outcomes, particularly postoperative adverse events, which require larger studies. Additionally, as a single-center study, its findings lack generalizability, necessitating multi-center research. Future studies should also examine tumor size to assess its impact on long-term prognosis.

Current evidence suggests that MSR-ESE is a safe and effective surgical technique. Compared to traditional ESE, MSR-ESE reduces operation time, hospital stay, and costs, while lowering the incidence of postoperative peritonitis, making it a safer and more efficient option.

## Data Availability

Publicly available datasets were analyzed in this study. The datasets used and analyzed during the current study are available from the corresponding author on reasonable request.
